# Anticancer activity of TTAC-0001, a fully human anti-vascular endothelial growth factor receptor 2 (VEGFR-2/KDR) monoclonal antibody, is associated with inhibition of tumor angiogenesis

**DOI:** 10.1080/19420862.2015.1086854

**Published:** 2015-09-01

**Authors:** Dong Geon Kim, Younggeon Jin, Juyoun Jin, Heekyoung Yang, Kyeung Min Joo, Weon Sup Lee, Sang Ryeol Shim, Sung-Woo Kim, Jinsang Yoo, Sang Hoon Lee, Jin-San Yoo, Do-Hyun Nam

**Affiliations:** 1 Institute for Refractory Cancer Research, Research Institute for Future Medicine, Samsung Medical Center; Seoul 06351, Korea; 2 Department of Health Sciences and Technology; SAIHST, Sungkyunkwan University; Seoul 06351, Korea; 3 Department of Neurosurgery; Samsung Medical Center, Sungkyunkwan University School of Medicine; Seoul 06351, Korea; 4 Department of Anatomy and Cell Biology; Sungkyunkwan University School of Medicine; Seoul 06351, Korea; 5 PharmAbcine; Inc., Daejeon Bioventure Town; Daejeon 34054, Korea; 6 Bio Division, Hanwha Chemical; Seoul 04541, Korea

**Keywords:** TTAC-0001, VEGF signaling, fully human neutralizing antibody, angiogenesis inhibitor, tumor growth inhibition, pharmacokinetics, glioblastoma, colorectal cancer

## Abstract

Vascular endothelial growth factor (VEGF) and its receptors are considered the primary cause of tumor-induced angiogenesis. Specifically, VEGFR-2/kinase insert domain receptor (KDR) is part of the major signaling pathway that plays a significant role in tumor angiogenesis, which is associated with the development of various types of tumor and metastasis. In particular, KDR is involved in tumor angiogenesis as well as cancer cell growth and survival. In this study, we evaluated the therapeutic potential of TTAC-0001, a fully human antibody against VEGFR-2/KDR. To assess the efficacy of the antibody and pharmacokinetic (PK) relationship in vivo, we tested the potency of TTAC-0001 in glioblastoma and colorectal cancer xenograft models. Antitumor activity of TTAC-0001 in preclinical models correlated with tumor growth arrest, induction of tumor cell apoptosis, and inhibition of angiogenesis. We also evaluated the combination effect of TTAC-0001 with a chemotherapeutic agent in xenograft models. We were able to determine the relationship between PK and the efficacy of TTAC-0001 through in vivo single-dose PK study. Taken together, our data suggest that targeting VEGFR-2 with TTAC-0001 could be a promising approach for cancer treatment.

## Abbreviations

ATCC: American Type Culture Collection
AUC_inf_: area under the serum concentration–time curve from time 0 extrapolated to infinity
AUC_last_: the area under the serum concentration–time curve from time 0 up to the last measurable concentration
C_L_: total body clearance
CD31: cluster of differentiation 31
GBM: glioblastoma multiforme
Hb: hemoglobin
i.p.: intraperitoneal
i.v.: intravenous
KDR: kinase insert domain receptor
LSD: least significant difference
MVD: microvessel density
PBS: phosphate-buffered saline
PCNA: proliferating cell nuclear antigen
PD: pharmacodynamics
PECAM-1: platelet endothelial cell adhesion molecule 1
PFS: progression-free survival
PK: pharmacokinetics
Q1W: once a week
T_1/2_: half-life
TUNEL: terminal deoxynucleotidyl transferase dUTP nick end labeling
V_ss_: volume of distribution at steady state
VEGF: vascular endothelial growth factor
VEGF-A: vascular endothelial growth factor A
VEGFR-1: vascular endothelial growth factor receptor 1
VEGFR-2: vascular endothelial growth factor receptor 2

## Introduction

Vascular endothelial growth factor (VEGF), one of the major angiogenic signal promoting molecules, and its receptor (VEGFR) play a crucial role in angiogenesis and lymphangiogenesis.^1,2^ According to recent reports, angiogenesis is a complex process regulated by numerous stimulatory and inhibitory factors. Despite the various molecules involved in angiogenesis, the VEGF/VEGFR pathway still continues to be the key regulator of the angiogenic process and is often highly expressed in various human cancers including colorectal cancer and glioblastoma.^3,4^

There are 5 forms of VEGF (VEGF-A, VEGF-B, VEGF-C, VEGF-D, PlGF) and 3 forms of the receptor (VEGFR1, VEGFR2, VEGFR3). VEGF-A, VEGF-B and PIGF bind to VEGFR1; VEGF-A, VEGF-C and VEGF-D bind to VEGFR2; VEGF-C and VEGF-D bind to VEGFR3, and the varied molecular interactions cause different biological responses.^5^ Among these growth factors, VEGF-A mediates development and maintenance of tumor vessel networks.^6^ Also, VEGFR-1 and VEGFR-2/kinase insert domain receptor (KDR) are involved in angiogenesis through binding with VEGFs due to their high affinity to the receptors. Of these 2 tyrosine kinase receptors, VEGFR-2 is the major signaling regulator of the VEGF/VEGFR pathway; thus, developing a therapeutic agent targeting VEGFR-2/KDR seems an optimal choice for anti-angiogenesis clinical intervention.^7^

Accumulating experimental and clinical studies indicate that uncontrolled angiogenesis is a major contributor to tumor progression.^8,9^ Therefore, inhibition of tumor angiogenesis is considered as an ideal strategy in cancer treatment. For this reason, various anti-angiogenesis inhibitors targeting VEGF signaling pathways have already been developed, including antibodies, immunotoxins, ribozyme, soluble receptors, and small molecule tyrosine kinase inhibitors.^10-12^ Among them, bevacizumab (Avastin®, Genentech), an anti-VEGF humanized monoclonal antibody that prevents VEGF from binding to VEGFR-1 and VEGFR-2, was approved for metastatic colon cancer.^13^ However, recent clinical trial results in glioblastoma and metastatic breast cancers proved to be disappointing and modest at best, suggesting that targeting the VEGF ligand may not be the best strategy in some tumors.^14,15^

TTAC-0001 is a novel human anti-VEGFR-2 monoclonal antibody generated by PharmAbcine, Inc.. that can inhibit VEGF/VEGFR-2 interaction.^16^ Our previous study has shown that TTAC-0001 binds specifically to the extracellular domain of human VEGFR-2 and prevents VEGFR from binding to VEGFR-2/KDR, which in turn inhibits its downstream signaling and the remodeling process of vessel formation.^17^ In this report, we show that TTAC-0001 effectively inhibits angiogenesis in various in vivo models, and also that the combination of TTAC-0001 with cytotoxic chemotherapy resulted in enhanced antitumor activity. Furthermore, pharmacokinetic (PK) studies were performed in mice to support the preclinical and clinical applications of TTAC-0001. Taken together, our data provide robust preclinical rationale and support for using TTAC-0001 as a novel clinical anti-angiogenesis therapy for cancer treatment.

## Results

### TTAC-0001 inhibits angiogenesis in vivo

**Figure 1.**
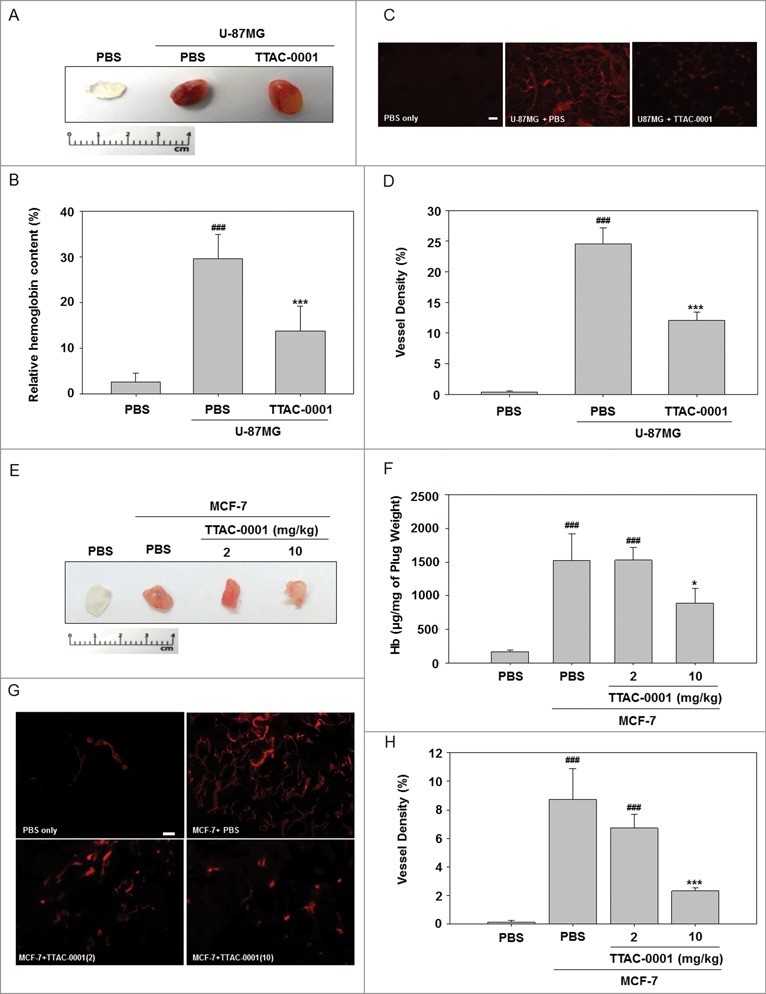
TTAC-0001 exhibits anti-angiogenic activity in U-87MG and MCF-7 Matrigel plug assays. Neovascularization in Matrigel plugs was quantified by evaluating hemoglobin (Hb) content after injecting female BALB/c-nu mice with 0.5 mL Matrigel mixed with 1 × 10^6^ U-87MG cells and 5 × 10^6^ MCF-7 cells into the bilateral flanks. Mice were treated with intravenous injection of 10 mg/kg TTAC-0001. Matrigel plugs with U-87MG cells were removed at day 10. (**A**) Gross overview of Matrigel plug and (**B**) hemoglobin (Hb) content (mean ± SE, *n* = 8). (**C**) Immunohistochemical images showing CD31-positive blood vessels (red) in the Matrigel plug. Scale bars = 200 µm. (**D**) Density of CD31-positive blood vessels in the Matrigel plug. (mean ± SE, *n* = 8). ^###^ *p* < 0.001 vs. phosphate buffered saline (PBS) only, *** *p* < 0.001 vs. U87MG + PBS. Matrigel plugs with MCF-7 cells were removed at day 10. (**E**) Gross overview of Matrigel plug and (**F**) Hb content (mean ± SE, *n* = 8). (**G**) Images showing CD31-positive blood vessels (red) in the Matrigel plug. Scale bars = 200 µm. (**H**) Densities of CD31-positive blood vessels in the Matrigel plug (mean ± SE, *n* = 8). * *p* < 0.05, *** *p* < 0.001 vs. MCF-7 + PBS.

To evaluate the efficacy of TTAC-0001 to inhibit angiogenesis in vivo, we used a Matrigel plug assay on both U-87MG (human glioblastoma cell line) and MCF-7 (human breast cancer cell line) tumor cells. VEGF secreted from U-87MG and MCF-7 cells exert pro-angiogenic effects in the Matrigel, thus promoting neovascular development. In both cellular models, Matrigel plugs from the TTAC-0001-treated groups were pale white in appearance, in contrast to the control group, which was bright red in both models, consistent with a reduction in new blood vessel formation **(Fig. 1A, 1E)**. Excised Matrigel plugs were collected and examined for hemoglobin (Hb) content. The Matrigel plugs with U-87MG or MCF-7 cells had higher Hb content than the Materigel plugs with phosphate buffered saline (PBS; *p* < 0.001). The hemoglobin (Hb) content in TTAC-0001-treated Matrigel plugs was significantly lower than that of the vehicle-treated Matrigel plugs (*p* < 0.05; **Fig. 1B, 1F**). The ability of TTAC-0001 to inhibit neovascularization was subsequently evaluated with anti-CD31 antibodies to quantify vessel density. In comparison with the PBS-treated group, the 10 mg/kg TTAC-treated group showed drastically reduced blood vessel densities (*p* < 0.05; **Fig. 1C, 1D, 1G, 1H**).

### TTAC-0001 has antitumor activity in human glioblastoma xenograft models

**Figure 2.**
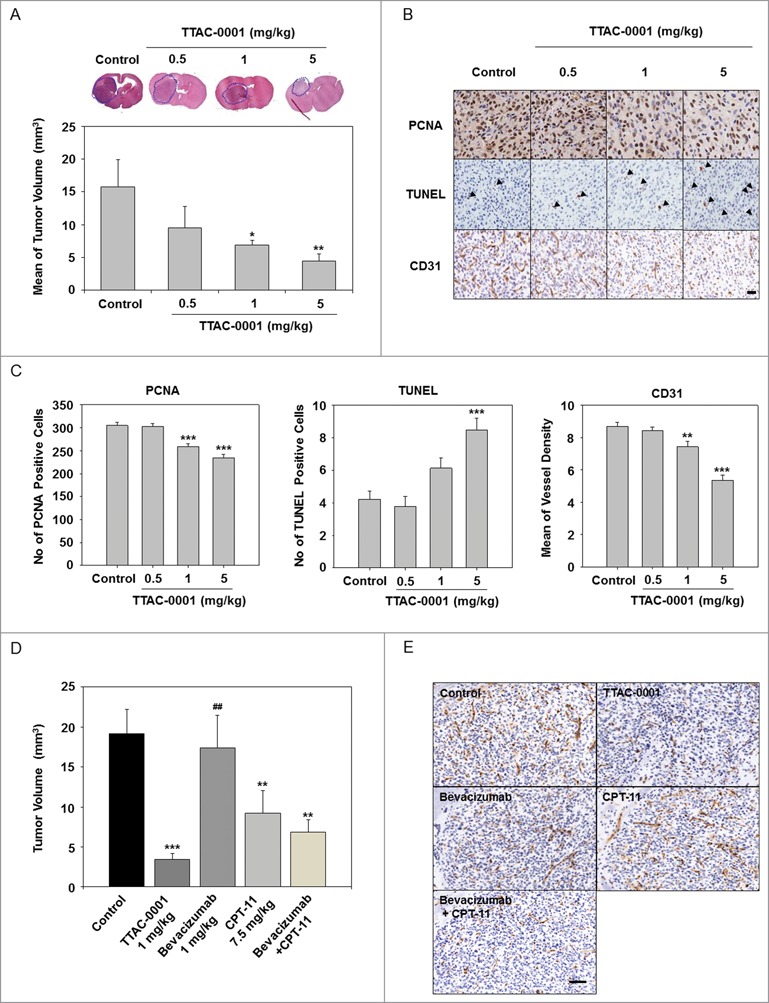
TTAC-0001 inhibits in vivo tumor growth in U-87MG xenograft models. (**A**) TTAC-0001 inhibits tumor growth in a U-87MG orthotopic xenograft model. Treatment groups exhibited significantly smaller tumor volumes (mean ± SE, *n* = 7) than control. (**B**) Paraffin embedded or frozen sections of the orthotopic tumors were stained for proliferating cells using anti-proliferating cell nuclear antigen (PCNA) antibody (upper panels), apoptotic cells using the terminal deoxynucleotidyl transferase dUTP nick end labeling (TUNEL) assay (middle panels), and endothelial cells using anti-CD31 antibody (lower panels), respectively (scale bar = 200 µm). (**C**) PCNA-positive cells, TUNEL-positive cells, and microvessel density were quantified. (**D**) In the U-87MG orthotopic glioblastoma models, TTAC-0001 (1 mg/kg) treatment resulted in better tumor growth inhibition than bevacizumab, CPT-11, or bevacizumab + CPT-11 combination treatment. (**E**) Paraffin sections of U-87 MG tumors were stained with anti-CD31 antibody. Scale bar = 200 µm. * *p*< 0.05, ** *p* < 0.01, and *** *p* < 0.001 vs. Control. ^##^ *p* < 0.001 vs. TTAC-0001 1 mg/kg.

To evaluate the antitumoral effects of TTAC-0001 in a glioblastoma orthotopic model, U-87MG human glioblastoma cell lines were inoculated into the caudate nucleus of BALB/c-nu mice. TTAC-0001 (0.5, 1, or 5 mg/kg) or vehicle was administered intravenously (i.v.), 14 d after the inoculation. TTAC-0001 treatment resulted in a dose-dependent reduction of tumor volume compared to the vehicle-treated group. Tumor growth rate were significantly inhibited in 1 or 5 mg/kg TTAC-0001-treated groups than control group. (*p* < 0.05 and *p* < 0.01, respectively, **Fig. 2A**). Body weight loss was not observed in the TTAC-0001-treated group throughout the study period (data not shown). Also, immunohistochemical analysis in tumor tissues demonstrated a significant reduction of proliferating cell nuclear antigen (PCNA) cells and microvessel density (MVD) along with a significant increase of terminal deoxynucleotidyl transferase dUTP nick end labeling (TUNEL)-positive apoptotic cells **(Fig. 2B, 2C)** by TTAC-0001 treatment.

The therapeutic effect of TTAC-0001 was also observed in U-87MG subcutaneous tumors. TTAC-0001 was injected i.v. once a week at 1 or 4 mg/kg and a significant inhibition in tumor volume (*p* < 0.05) was seen after this treatment when compared to the control group **(Fig. S1)**.

Based on a recent Phase 2 clinical trial where glioblastoma patients, treated with a combination of bevacizumab and CPT-11, showed an increased median overall survival rate (approximately 9.7 months), we also compared the antitumor effects of TTAC-0001 in U-87MG glioblastoma models with combination of bevacizumab and CPT-11 treatment.^18^ As shown in **Figure 2D**, a significant decrease in tumor volume was observed in the TTAC-0001- and the CPT-11 monotherapy groups compared to that in the control group. Although no specific changes were observed in bevacizumab monotherapy, the combination therapy of bevacizumab + CPT-11 showed a decreased tumor volume (*p* < 0.01) similar to that in the clinical study. Interestingly, the TTAC-0001-treated group showed the most effective reduction of tumor volume compared to those treated with bevacizumab, CPT-11 or combined bevacizumab + CPT-11. To determine the antiangiogenic effects of TTAC-0001, U-87MG tumor sections were immunostained with CD31 antibody, an endothelial cell marker **(Fig. 2E)**. The number of CD31-positive cells was reduced in tumors treated with bevacizumab in combination with CPT-11 relative to that in the tumors with bevacizumab or CPT-11 monotherapy. In addition, CD31-positive cells were drastically reduced in the TTAC-0001-treated tumors compared to the bevacizumab, CPT-11 or the combination group.

### TTAC-0001 suppresses tumor growth in human colorectal cancer xenograft models

**Figure 3.**
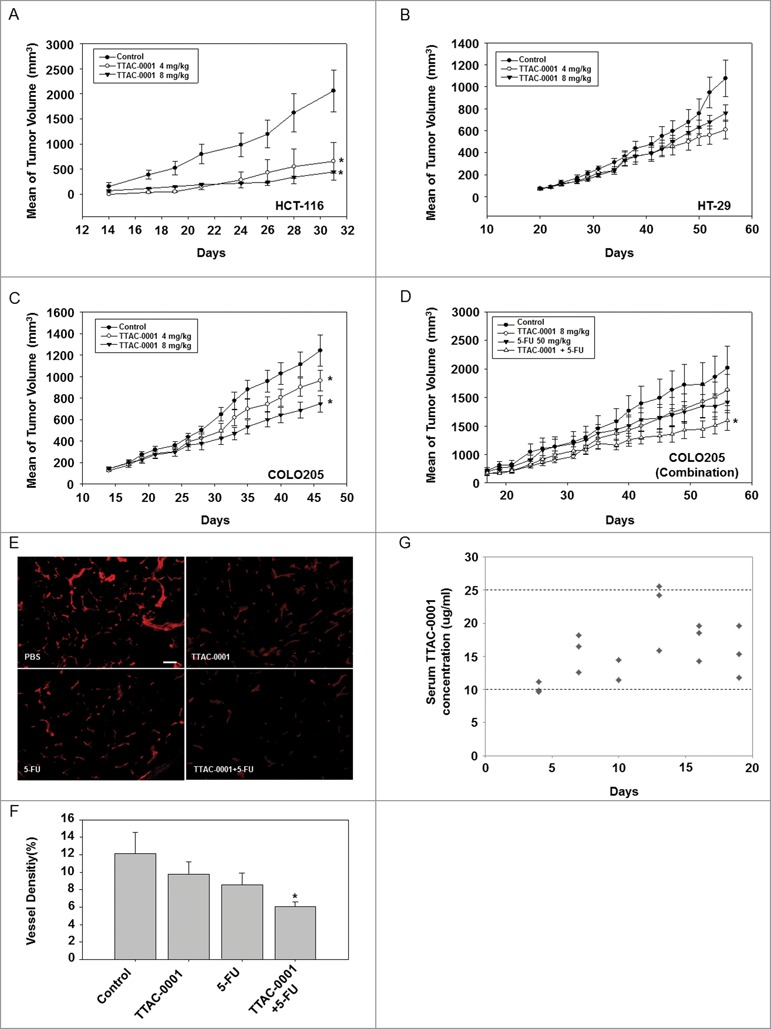
TTAC-0001 inhibits tumor growth of transplantable human colon cancer cells in BALB/c-nu mice. Tumor growth curves of transplantable human colon carcinomas in female BALB/c-nu mice. (**A−C**) Mice were inoculated subcutaneously in the right flank with human colon cancer cells HCT-116, COLO205, or HT-29. Phosphate-buffered saline (PBS) or 4 or 8 mg/kg TTAC-0001 were injected intravenously once per week. Tumor sizes were measured 3 times per week using a caliper (mean ± SE, *n* = 8). (**D**) Combination treatment of TTAC-0001 and 5-fluorouracil (5-FU) leads to greater tumor volume reduction. Mice were given injections of PBS, TTAC-0001, 5-FU, or 5-FU + TTAC-0001 (Mean ± SE, *n* = 8). * *p*< 0.05 vs. control. (**E**) Immunohistofluorescence analysis of CD31-positive blood vessels (red) in the xenograft tumor. Scale bars = 200 µm. (**F**) Densities of CD31-positive blood vessels in the xenograft tumor (VD = vessel density; *n* = 8; * *p* < 0.05 vs. control). (**G**) Relationship between serum TTAC-0001 concentration and efficacy in COLO205 tumors. TTAC-0001 concentrations were measured in serum every 3 d. Mice were intravenously administered 8 mg/kg TTAC-0001, once per week.

In vivo antitumoral effects of TTAC-0001 were also evaluated in colorectal cancer xenograft models (HCT116, HT29, and COLO205). TTAC-0001 (4 or 8 mg/kg) was given i.v. once per week. Dose-dependent tumor growth inhibition (30−86%) by TTAC-0001 was observed in all 3 cellular models. No significant toxicity, which was evaluated according to body weight changes, was observed during the experiments (data not shown). TTAC-0001 treatment at 4 or 8 mg/mL resulted in a significant reduction of tumors in the HCT116 (*p* < 0.05) and COLO205 (*p* < 0.05) xenograft models **(Fig. 3A, 3C)**, whereas only moderate inhibition of tumor growth was observed in the HT29 xenograft model (**Fig. 3B**). We also evaluated the effects of combination treatment with chemotherapy and TTAC-0001. COLO205 tumor xenograft models were treated with TTAC-0001 (8 mg/kg, i.v., every 3 days), 5-fluorouracil (5-FU; 50 mg/kg, i.p., once per week), or combined TTAC-0001 + 5-FU. TTAC-0001 or 5-FU monotherapy inhibited tumor growth in the COLO205 xenograft models at a similar rate. However, the combination treatment group showed higher tumor growth inhibition than the single agent treatment groups and also the control group (*p* < 0.05) **(Fig. 3D)**. The ability of TTAC-0001 to inhibit new blood vessel formation was subsequently evaluated with anti-CD31 antibody through quantification of MVD **(Fig. 3E, 3F)**. As shown in **Figure 3F**, TTAC-0001 or 5-FU monotherapy only slightly reduced the microvessel development whereas the combination groups significantly reduced the microvessel development (*p* < 0.05). Body weight loss was not observed in the combination treatment group throughout the study (data not shown).

### Pharmacokinetic and pharmacodynamic relationship of TTAC-0001 in human colorectal cancer xenograft models

To evaluate the pharmacokinetic (PK)/ pharmacodynamic (PD) relationships in this study, the correlation between efficacy and serum concentrations of TTAC-0001 was investigated in COLO205 colon tumor-bearing athymic mice. In COLO205 subcutaneous xenograft models **(Fig. 3C, 3D)**, mice were administrated TTAC-0001 (8 mg/kg, i.v.) once per week after the tumor volumes reached 200 mm^3^. The total clearance rate was determined by the serum concentration level of TTAC-0001 in mice. As shown in **Figure 3G**, serum concentrations of TTAC-0001 were maintained between 10–25 μg/mL throughout the experiment (for 21 days). Our results show the expected concentration rate of TTAC-0001 in mouse serum when the mice were administrated 8 mg/kg of TTAC-0001, the effective dosing level for tumor growth inhibition in COLO205 xenograft models. Furthermore, our data suggest that maintenance of the serum concentration of TTAC-0001 (10-25 μg/mL) may be responsible for determining the efficacy of TTAC-0001. Taken together, a TTAC-0001 concentration of 10 µg/mL or above is recommended in order to achieve optimal results in vivo.

### PK of TTAC-0001 in BALB/c mice and BALB/c-nu mice

**Figure 4.**
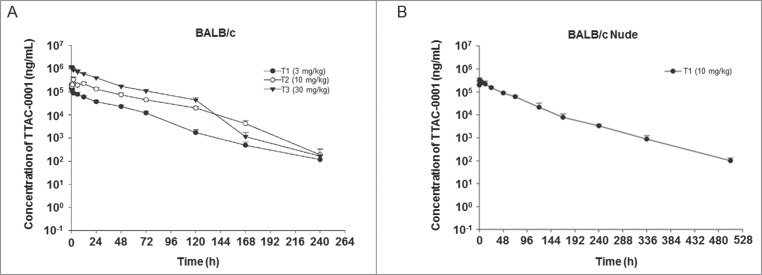
Pharmacokinetic analysis of single-dose TTAC-0001 treatment. Profile of serum TTAC-0001 concentration vs. time in mice given an intravenously administered single dose of TTAC-0001. At the indicated times after dosing, serum samples were obtained from caudal vena cava of individual mice, and the concentration of inhibitor in each sample was determined by enzyme-linked immunosorbent assay. These tests were performed in (**A**) BALB/c mice and (**B**) BALB/c-nu mice. Mean ± SE.

The PK of TTAC-0001 was studied after a single i.v. dose of 3, 10, or 30 mg/kg (*n* = 3 mice/group) in BALB/c mice and 10 mg/kg (*n* = 3 mice/group) in BALB/c-nu mice. The increase in the area under the serum concentration–time curve (AUC) from time 0 up to the last measurable concentration (AUC_last_) for TTAC-0001 treatment was dose-proportional **(Fig. 4A, 4B)**. Values for the AUC_last_ for 3, 10, and 30 mg/kg of TTAC-0001 (1:3:10 ratios) were increased at 1:4:10 ratios, respectively. This suggests that PK is represented by a linear model in mouse models within the tested dose range **(Table S1)**. Half-life (t_1/2_), total body clearance (C_L_), and distribution volume at steady state (V_ss_) in BALB/c mice were approximately 19–36 hours, 1 mL/h/kg, and 31–37 mL/kg, respectively. Furthermore, the PK profiles in BALB/c-nu mice were similar to BALB/c mice, but t_1/2_ was slightly higher, as it increased to 55 h from 19−36 h.

## Discussion

Angiogenesis is a critical step in tumor development and metastasis as tumor cells maintain their continuous growth by obtaining sufficient oxygen and nutrients from blood vessels.^19-21^ The importance of VEGF/VEGFR-2 signaling in endothelial cell function in tumors is well established. For example, various preclinical studies have shown that treatment with bevacizumab (anti-VEGF antibody) or DC-101 (anti-mouse VEGFR-2 antibody) showed strong efficacy in many xenograft models by suppression of tumor angiogenesis.^6,22^

Recently, ramucirumab, a fully human IgG1 monoclonal antibody that inhibits VEGFR-2/KDR, was approved in the US as a monotherapy for advanced or metastatic gastric cancer or gastro-esophageal junction adenocarcinoma.^23,24^ Previous studies have suggested that targeting VEGFR-2/KDR has more advantages than targeting the VEGF ligand itself as: (1) In hypoxic tumor microenvironments, other VEGF-like heparin-binding growth factors that localize in the extracellular matrix of tissues can prevent specific inhibition by the anti-VEGF antibody, (2) VEGF is relatively inaccessible to drugs because it is mainly localized in the interstitial space of tissues,^25,26^ while VEGFR-2/KDR is more easily accessible as it is highly expressed on the surface of the activated endothelium in tumor tissues, (3) VEGFR-2 inhibition suppresses the effects of VEGF-A, -C and -D, whereas VEGF antagonists such as bevacizumab only inhibit VEGF-A activity,^27^ and (4) in particular, therapeutic antibodies targeting VEGFR-2, especially those of the human class IgG1, may be more efficient in killing targeted cells via immune effector signaling cascades like antibody-dependent cell-mediated cytotoxicity or complement-dependent cellular cytotoxicity.^28^ This approach would exploit the “leakiness” of blood-tumor barriers in glioblastoma, which often possess disrupted blood-brain barriers due to tumor burden.^29,30^

Our previous study demonstrated that TTAC-0001 binds specifically to the N-terminus of domain 2 and domain 3 in the extracellular region of KDR and competitively inhibits the binding of VEGF-C and VEGF-D to KDR. The Ig domain 2 and 3 share highly similar epitopes between human and murine species, therefore, cross-reactive activity of TTAC-0001 has been observed between these species.^17^ The specific binding affinity and the cross reactivity of TTAC-0001 offer an opportunity to evaluate the therapeutic efficacy with studying its predictive PK characteristics in preclinical models.

Here, we report data for TTAC-0001, a novel fully human anti-VEGFR-2/KDR monoclonal antibody that blocks VEGF/VEGFR-2 signaling. According to our previous study, TTAC-0001 interrupted the downstream signaling of VEGF/KDR and induced anti-angiogenic effects in vitro without antibody-dependent and cell-mediated cytotoxicity or complement-dependent cellular cytotoxicity.^17^ In this study, we show the antitumoral efficacy of TTAC-0001 in various xenograft tumor models, along with the predictive PK characteristics in vivo. Also, TTAC-0001 potently inhibited in vivo vasculogenesis and angiogenesis. Inhibitory effects of TTAC-0001 on angiogenesis were determined consistently in various angiogenesis assays.

Treatment with TTAC-0001 significantly inhibited CD31-positive MVD in U87MG and MCF7 Matrigel plug assays. These results are further supported by the fact that necrosis and tumor growth inhibition in U-87MG models were observed in the diffusion-limited region immediately surrounding the surviving vasculature. In addition, TTAC-0001 induced robust antitumor activity as a single agent in U87MG xenograft tumors, which express high levels of mouse VEGFR-2.^31^ Therefore, these results suggest that TTAC-0001 effectively blocks VEGF/VEGFR-2 signaling and inhibits angiogenesis in mouse xenograft and orthotopic models.

In vivo efficacy of TTAC-0001 was evaluated in various colorectal xenograft models. In these studies, TTAC-0001 consistently inhibited tumor growth by 30−86%. Interestingly, it was reported that xenograft tumors that highly express mouse VEGFR-2 were more prone to tumor growth inhibition by anti-VEGF antibody treatment.^32^ However, according to the studies that were conducted on A549, TTAC-0001 was able to show antitumor activity in low VEGFR-2/KDR expressing lung cancer xenograft models as well. The inhibitory effect of tumor growth was due to targeting VEGFR-2 in the host mouse endothelial cells and the tumor microenvironments (**Fig. S2**). Taken together, these results are consistent with the notion that VEGFR-2 functions as a key target in tumor angiogenesis; selective inhibition of VEGFR-2 may translate into an effective anti-angiogenesis therapy.

To evaluate in vivo efficacy of combination treatment with TTAC-0001 and chemotherapeutic agents, we utilized a COLO205 xenograft model. We demonstrated that TTAC-0001 could positively cooperate with other chemotherapy agents, such as 5-FU, to inhibit tumor growth in colorectal models, resulting in decreased tumor MVD. Considering previous clinical trial results for angiogenesis inhibitors with chemotherapeutic agents, and our preclinical data of TTAC-0001, there may be substantial opportunities to improve response rates and extend progression-free survival (PFS).^33-36^

Glioblastoma multiforme (GBM) expresses extremely high levels of VEGF and is characterized as a highly vascularized tumor.^37,38^ Molecular profiling of glioblastoma tumors indicates strong expression of VEGF and its receptor, VEGFR-2/KDR. A subset of GBMs characterized by high levels of microvascular proliferation correlates with poor patient prognosis.^39-41^ In the Phase 3 clinical trial of bevacizumab for relapsed disease, treatment with this antibody reduced tumor volume and frequency of edema, increasing PFS and median overall survival to 6 and 9.3 months, respectively.^42^ We utilized subcutaneous and orthotopic xenograft mouse models of human GBM cell line U-87MG to directly monitor in vivo antitumoral effects of TTAC-0001 and bevacizumab. In this study, TTAC-0001 significantly inhibited tumor growth and extended survival rate. Through immunohistochemistry analysis, we found that TTAC-0001 significantly inhibited proliferation of tumor cells and endothelial cells, suggesting that TTAC-0001 suppresses the VEGF/VEGFR-2 pathway in the tumor microenvironment and could be utilized in the clinic for GBM patient treatment.

A major hurdle to be overcome in the development of effective therapies for GBM is the difficulty of delivering a drug into the brain. The blood-brain barrier effectively blocks most proteins and many pharmacologic agents from passing through into the brain.^43^ However, the leakiness of GBM blood vessels allows the systematic delivery of bevacizumab into the affected regions of the brain, providing even more clinical benefits than other agents.

In addition, a previous clinical trial that combined treatment of bevacizumab + CPT-11 showed excellent penetration through the blood-brain barrier.^18^ These trials showed the combination effect of these 2 treatments, which resulted in response rates between of 44% and 57% and median overall survival of 9.7 months. Therefore, these results suggest that systemic deliverance of antibodies, such as TTAC-0001, may provide more benefits than bevacizumab monotherapy or the combination treatment of bevacizumab with CPT-11 in GBM, due to direct targeting of VEGFR-2/KDR in tumors through leaky vessels.

To better understand the PK parameters in mice, we modeled TTAC-0001 clearance in BALB/c and BALB/c-nu mice. Even though direct interference effects of mouse IgG in the PK analysis was not measured, we have demonstrated that there was little difference between the with/without mouse serum condition (data not shown). Our data further showed that there was no interference by the irrelevant IgG in the serum. According to the PK study results, the predictable levels of AUC from time 0 extrapolated to infinity (AUCinf) of whole body-exposure were similar between the BALB/c and BALB/c-nu mice, albeit the half time was slightly increased in the BALB/c-nu mice compared to the BALB/c-mice when TTAC-0001 was administered (i.v. 10 mg/kg once).

Therapeutic monoclonal antibodies typically show pharmacological characteristics such as small volume of distribution (< 3−5L), low rate of clearance (0.5−4 mL/min/kg) and a diverse biological t_1/2_ between 2 and 24 d in humans. Our results indicate that TTAC-0001 possesses a shorter t_1/2_ (36 h in BALB/c, 52 h in BALB/c-nu) and more rapid rate of total clearance (1 mL/h/kg in BALB/c, 0.8 mL/h/kg in BALB/c-nu) than numerous monoclonal antibodies that are currently approved for marketing. ^44,45^ However, the reasons for the rapid clearance of TTAC-0001 in the plasma and its elimination mechanism remain unclear.

Interestingly, although TTAC-0001 was rapidly eliminated in mice, we were able to ascertain therapeutic efficacies in various preclinical models in this study. Notably, the steady-state concentration of TTAC-0001 was maintained between 10 and 25 μotablin the COLO205 xenograft model, which was in a similar range of maximum biological activity to TTAC-0001 in vitro (5–20 μg/ml).^17^ Based on in vitro and in vivo data, it was determined that the minimum serum concentration (Cmin) to achieve pharmacological activity should be higher than 20 ug/mL. Based on preclinical PK data of our study, the suggested dose for Phase 1 clinical study should range over 5 mg/kg on a weekly schedule (Q1W) to achieve a target exposure of Cmin at steady state (20 μg/mL).

In summary, this study provides various preclinical data for TTAC-0001, a novel fully human monoclonal antibody against VEGFR-2/KDR, as an effective anti-angiogenic therapy. As a clinical approach, this research provides a critical observation for mono-/combination therapeutic efficacy of TTAC-0001 and its PK profile in animal models. Our study supports the feasibility of TTAC-0001 providing therapeutic benefits in glioblastoma and colorectal cancer through inhibitory effects of tumor-associated angiogenesis by preventing VEGF from binding to VEGFR-2/KDR. Additionally, the PK profiling study showed that TTAC-0001 may not remain in mouse tissues for a long time, while therapeutic effects were observed in preclinical models. Our results suggest that TTAC-0001 could be a promising drug to be used in a clinical trial for glioblastoma and colorectal cancer. The predicted human T_1/2_ and reduced immunogenicity profile associated with TTAC-0001 may offer distinct clinical advantages, and provide options for use as either a single agent or in combination with other chemotherapeutic agents for the treatment of solid tumors.

## Materials and Methods

### Cells and animals

Human glioblastoma cell line U87MG, human colorectal adenocarcinoma cell lines HCT116, HT-29, COLO205, and human breast cancer cell line MCF-7 were purchased from the American Type Culture Collection (ATCC). The cell lines were cultured under standard conditions recommended by the ATCC. BALB/c and BALB/c-nu mice (female, 6–9 weeks) were used for the in vivo studies. Animals were obtained from Orient Bio Inc., and were maintained under specific pathogen-free conditions in facilities approved by the Association for Assessment and Accreditation of Laboratory Animal Care International in accordance with the current regulations and standards of the Laboratory Animal Research Center at the Samsung Biomedical Research Institute.

### Pharmacokinetics

The PK of TTAC-0001 was evaluated in BALB/c and BALB/c-nu mice. By tail vein injection, 3, 10, or 30 mg/kg TTAC-0001 was administered to BALB/c mice (*n* = 3), and 10 mg/kg TTAC-0001 to BALB/c-nu mice (*n* = 3). Blood samples were collected from each mouse via the caudal vena cava at 0 min, 15 min, 30 min, 1 h, 2 h, 6 h, 12 h, 1 day, 2 days, 3 days, 5 days, 7 days, 10 days, 14 days, and 21 d after the injection. The collected blood samples were incubated at room temperature for approximately 30 min. Serum concentrations of TTAC-0001 were measured by enzyme linked immunosorbent assay. To measure the serum concentrations of TTAC-0001, each well in the Maxisorp 96-well microplate was coated with 100 µL 2.5 μg/mL recombinant KDR-extracellular domain (1–3) (Pharmabcine, Korea) in 1x phosphate-buffered saline (PBS), pH 7.4 solution (Welgene, Korea), incubated at 4°C for more than 12 h, and washed with PBS/0.05% Tween-20 solution 3 times. And then, the plate was incubated with 200 μL of a blocking buffer (1x PBS, 0.5% BSA, 0.05% tween 20, 0.05% Proclin300, 0.25% CHAPS, 0.2% BGG, 5 mM EDTA, 0.35 M NaCl, pH 8.0) for 1 h. After washing the plate, assay calibrators were prepared by serial dilution of TTAC-0001 in BALB/c mouse blank serum (Biochemed Services). The calibration curve points were 0.98, 1.95, 3.91, 7.81, 15.6, 31.3, 62.5, 125, 250, 500, and 1,000 ng/mL. Anchor points (0.98, 1.95, and 1,000 ng/mL) that were outside the range of quantification were included or excluded to properly obtain the quality controls. The quality controls were also prepared by diluting the TTAC-0001 with BALB/c mouse blank serum. The level of LLOQ (lower limit of quantification) was 3.91 ng/mL, low quality control was 9.3 ng/mL, middle quality control was 44 ng/mL, high quality control was 233 ng/mL, and ULOQ (upper limit of quantification) was 500 ng/mL. The serum samples were diluted from 1 to 50,000 folds in BALB/c mouse blank serum. Then, all prepared calibrators, quality controls, serum blanks and samples were diluted with a 1:10 minimum dilution in a dilution buffer (1x PBS, 0.5% BSA, 0.05% tween 20, 0.05% Proclin300, 0.25% CHAPS, 5 mM EDTA, 0.6 M NaCl, pH 8.9). 100 μL of each component was aliquoted into each well in duplicates and incubated at 25°C for 2 h. After washing, the plate was incubated with 100 μL of detection antibody solution, (i.e., horseradish peroxidase-conjugated goat anti-human IgG (heavy and light chains) antibody (Bethyl laboratories) that was diluted 1:2,000 ratio in PBS/0.05% Tween-20 solution, at 25°C for 2 h in dark conditions. After washing with PBS/0.05% Tween-20 solution 5 times, the plate was incubated with 100 μl of freshly mixed 3,3′,5,5′-tetramethylbenzidine chromogenic solution (BD Biosciences) and incubated at 25°C for 5−20 min. Once the color changes were observed, the reaction was stopped by adding 50 μL of 2N H_2_SO_4_ stop solution to each well. Optical density was measured at 450 nm using a microplate reader (Tecan), and the data were processed using the Softmax Pro software. Serum concentrations of TTAC-0001 were interpolated from a 4-parameter logistic fit of the standard curve on the same plate. Non-compartmental PK parameters were calculated using WinNonlin (Pharsight) software package.

### Matrigel plug angiogenesis assay

The in vivo effect of TTAC-0001 on the inhibition of angiogenesis was evaluated using Matrigel plug angiogenesis assays with U-87MG or MCF-7 cells. Matrigel (0.5 mL) was premixed with 3 × 10^6^ U-87MG cells or 5 × 10^6^ MCF-7 cells before subcutaneous implantation into the bilateral flanks of BALB/c-nu mice (*n* = 8 per group). A single 2 or 10 mg/kg treatment of TTAC-0001 or vehicle was administered to the respective groups by i.v. injection after implantation. After 10 days, Matrigel plugs were removed and frozen for immunohistochemical analysis. To measure the Hb content, excised plugs (*n* = 8 plugs/group) were cut into small pieces and placed in 500 µL of cold, distilled water at 4°C overnight in order to liquefy the Matrigel. Specimens were centrifuged at 1,500 rpm for 20 min, and the supernatant was collected.^46^ Hb content was quantified with Drabkin's reagent kit (Sigma-Aldrich) and spectrophotometry. For microvessel counting studies, cryosections (30 µm) were stained with anti-mouse CD31 monoclonal antibodies; immunofluorescence images were analyzed to quantify MCD using ImageJ, a Java-based image processing and analysis program.

### Therapeutic efficacy of TTAC-0001 in various subcutaneous xenograft mouse models

The efficacy of TTAC-0001 was evaluated in various tumor xenograft models. HCT-116, HT-29, COLO205, and U-87MG cells were harvested, suspended in Hank's Balanced Salt Solution (HBSS), and subcutaneously injected into the flanks of BALB/c-nu mice (1 × 10^7^ or 3 × 10^6^ per mouse in 0.05 mL). When tumors reached approximately 200 mm^3^, animals were assigned into groups based on tumor volume to minimize intragroup and intergroup variation. After grouping, TTAC-0001 (1, 4, or 8 mg/kg) or vehicle were administered i.v. once per week. To analyze PK characteristics with the efficacy study, TTAC-0001 was administrated (8 mg/kg, i.v., once per week) in the COLO205 xenograft models. Blood samples were collected from each mouse, and the detailed process of PK analysis is described in the corresponding materials and methods section.

To evaluate the efficacy of combination therapy with 5-FU in COLO205 xenograft models, TTAC-0001 (8 mg/kg, i.v., once per week), 5-FU (50 mg/kg, i.p., once per week), TTAC-0001 + 5-FU, or vehicle were administered to the mice. Body weight was assessed and tumor diameters were measured using Vernier calipers. Tumor volumes were determined by calculating the volume of an ellipsoid using the formula: length [mm] ×o(width [mm]) ^2^ × 0.5). Tumor tissues were embedded in Optimal Cutting Temperature (OCT) compound, frozen rapidly in liquid nitrogen, and stored at −70°C. For counting the number of microvessels in the tumors, cryosections (30 µm) were stained with anti-mouse CD31 monoclonal antibodies; immunofluorescence images were analyzed to quantify MVD using ImageJ program.

### In vivo therapeutic efficacy in U-87MG orthotopic xenograft mouse model

U-87MG (2 × 10^5^ cells/5 μL HBSS) was intracranially injected into the white matter (coordinates: 0.5 mm anterior, 1.7 mm lateral, 3.2 mm depth from the bregma) with a rodent stereotactic device (Kopf instruments).^47^

For treatment with TTAC-0001 in the U-87MG models, the mice were randomized into 4 groups (*n* = 10 per group). TTAC-0001 (0.5, 1, or 5 mg/kg) or vehicle were administered i.v. at 14 d post-intracranial tumor-cell inoculation. To compare the efficacy of each treatment, TTAC-0001 (1 mg/kg, i.v., once per week), CPT-11(7.5 mg/kg, i.v., twice per week), bevacizumab (1 mg/kg, i.v. twice per week), bevacizumab + CPT-11 combination, or vehicle were administered. Twenty-eight days after the tumor cell inoculation, mice were anesthetized and euthanized. The brains of these mice were removed and sectioned axially. Sections were fixed in 10% buffered formalin and embedded in paraffin, and the other was embedded in OCT compound, frozen in liquid nitrogen, and stored at −70°C. The tumor volumes in the brain were calculated by measuring the section with the largest tumor portion and applying the previously described formula.^48^

### Immunohistochemistry and quantification of immunostains

For the detection of PCNA, TUNEL, and CD31 in the tumors, immunohistochemistry was performed as described previously.^48^ For PCNA, the sections were stained with mouse anti-PCNA (Dako). The DeadEnd™ fluorometric TUNEL system (Promega) was used to assay apoptosis. To quantify immunostaining for PCNA and TUNEL, the numbers of PCNA and TUNEL positive cells were counted in random fields at 400× magnification. The MVD was determined by immunohistochemistry using rat anti-mouse CD31/PECAM-1 antibodies (BD Pharmingen); stained cells were counted in random fields at 200× magnification.

### Statistics

Statistical comparisons of tumor regression were analyzed by one-way analysis of variance (ANOVA) followed by the least significant difference (LSD) test. Data are presented as mean ± SE. Kaplan-Meier estimates of survival function were plotted, and the statistical significance of differences in overall survival were calculated by the Mantel-Cox log-rank test. A significance level of *p* < 0.05 was used for all tests. PASW statistics software version 18.0 was used for all statistical analyses.
