# Supplementary material for: Anticancer activity of TTAC-0001, a fully human anti-vascular endothelial growth factor receptor 2 (VEGFR-2/KDR) monoclonal antibody, is associated with inhibition of tumor angiogenesis
Source: MAbs. 2015 Sep 1;7(6):1195–204. doi: 10.1080/19420862.2015.1086854 (PMC4966428; doi:10.1080/19420862.2015.1086854)
Supplement: Supplemental_Material.zip [file kmab-07-06-1086854-s001.zip › Supplemental Table S1.docx]

**Supplementary Table S1. Pharmacokinetic parameters of TTAC-0001 in mice after a single i.v. bolus dose.**

AUC = area under the serum concentration−time curve; CL = clearance;

t_1/2_= half‑life; V_d_ = volume of distribution; V_ss_ = volume of distribution at steady state

**
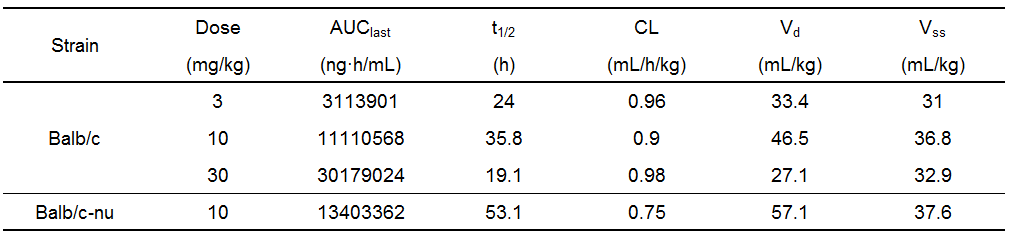
**
