# Supplementary material for: Anticancer activity of TTAC-0001, a fully human anti-vascular endothelial growth factor receptor 2 (VEGFR-2/KDR) monoclonal antibody, is associated with inhibition of tumor angiogenesis
Source: MAbs. 2015 Sep 1;7(6):1195–204. doi: 10.1080/19420862.2015.1086854 (PMC4966428; doi:10.1080/19420862.2015.1086854)
Supplement: Supplemental_Material.zip [file kmab-07-06-1086854-s001.zip › Supplemental Figure S1.pptx]

## Slide 1
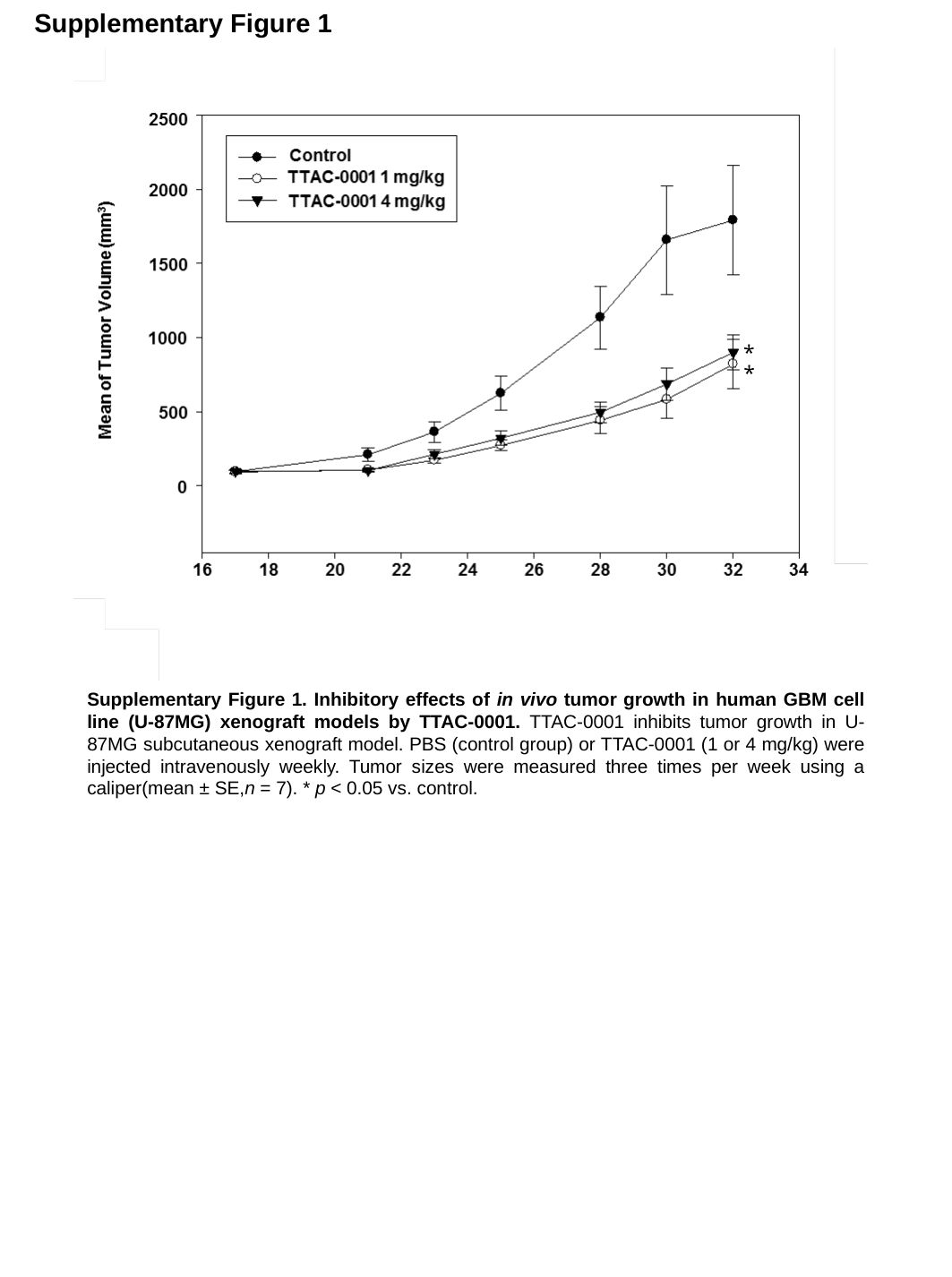

Supplementary Figure 1
*
*
Supplementary Figure 1. Inhibitory effects of in vivo tumor growth in human GBM cell line (U-87MG) xenograft models by TTAC-0001. TTAC-0001 inhibits tumor growth in U-87MG subcutaneous xenograft model. PBS (control group) or TTAC-0001 (1 or 4 mg/kg) were injected intravenously weekly. Tumor sizes were measured three times per week using a caliper(mean ± SE,n = 7). * p < 0.05 vs. control.
