# Supplementary material for: Anticancer activity of TTAC-0001, a fully human anti-vascular endothelial growth factor receptor 2 (VEGFR-2/KDR) monoclonal antibody, is associated with inhibition of tumor angiogenesis
Source: MAbs. 2015 Sep 1;7(6):1195–204. doi: 10.1080/19420862.2015.1086854 (PMC4966428; doi:10.1080/19420862.2015.1086854)
Supplement: Supplemental_Material.zip [file kmab-07-06-1086854-s001.zip › Supplemental Figure S2.pptx]

## Slide 1
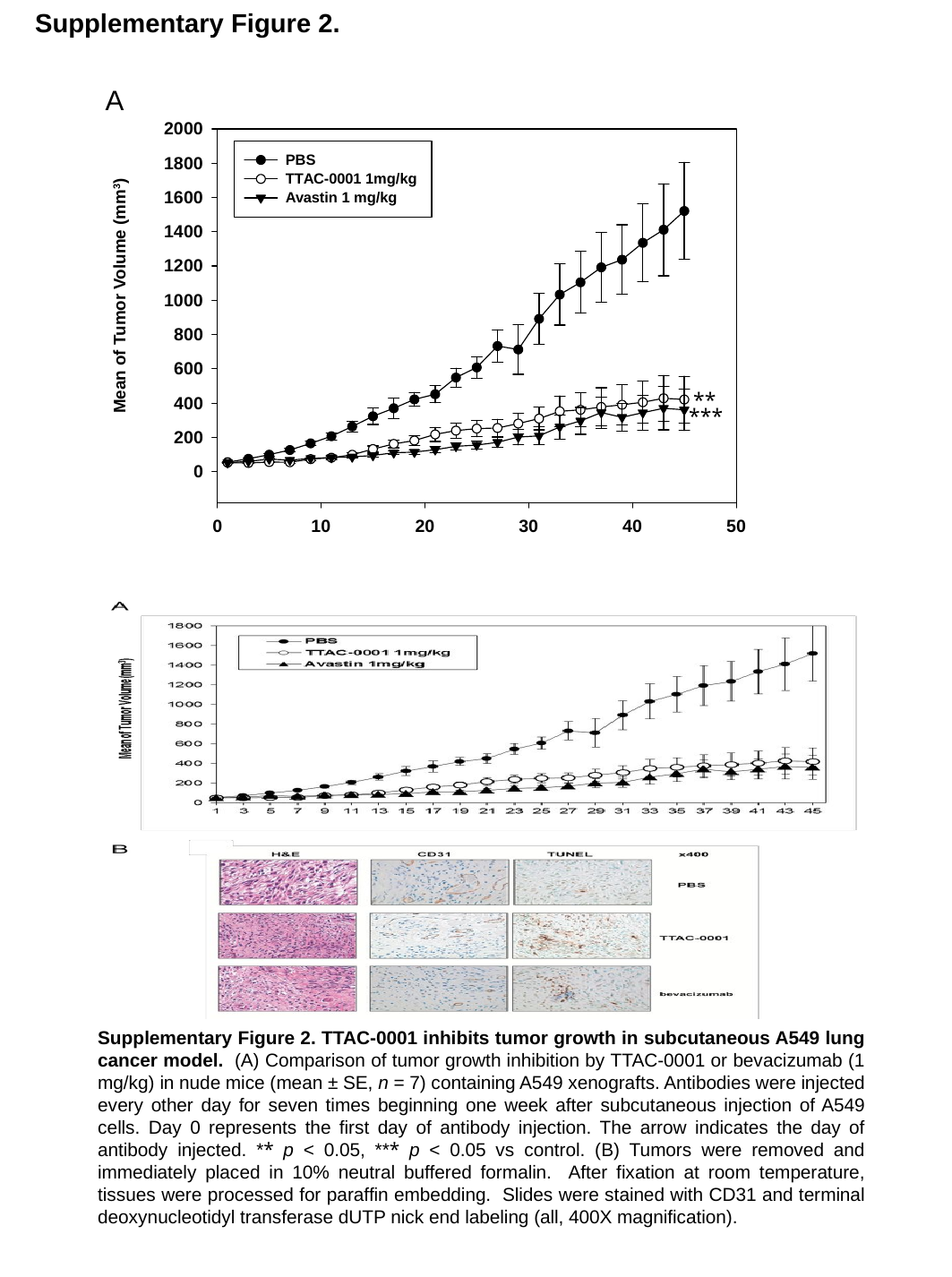

Supplementary Figure 2.
A
Mean of Tumor Volume (mm3)
**
***
Supplementary Figure 2. TTAC-0001 inhibits tumor growth in subcutaneous A549 lung cancer model. (A) Comparison of tumor growth inhibition by TTAC-0001 or bevacizumab (1 mg/kg) in nude mice (mean ± SE, n = 7) containing A549 xenografts. Antibodies were injected every other day for seven times beginning one week after subcutaneous injection of A549 cells. Day 0 represents the first day of antibody injection. The arrow indicates the day of antibody injected. ** p < 0.05, *** p < 0.05 vs control. (B) Tumors were removed and immediately placed in 10% neutral buffered formalin. After fixation at room temperature, tissues were processed for paraffin embedding. Slides were stained with CD31 and terminal deoxynucleotidyl transferase dUTP nick end labeling (all, 400X magnification).
